# The real world effect of omalizumab add on therapy for patients with moderate to severe allergic asthma: The ASTERIX Observational study

**DOI:** 10.1371/journal.pone.0183869

**Published:** 2017-08-31

**Authors:** Mohit Bhutani, William H. Yang, Jacques Hébert, Frederica de Takacsy, Jean-Louis Stril

**Affiliations:** 1 Department of Medicine, University of Alberta, Edmonton, Alberta, Canada; 2 Ottawa Allergy Research Corporation, University of Ottawa, Ottawa, Ontario, Canada; 3 Clinique Spécialisée en Allergie de la Capitale, Quebec City, Quebec, Canada; 4 Novartis Pharmaceuticals Canada Inc., Montreal, Quebec, Canada; Forschungszentrum Borstel Leibniz-Zentrum fur Medizin und Biowissenschaften, GERMANY

## Abstract

**Background:**

Omalizumab is a non-steroidal medication indicated for the treatment of poorly controlled moderate-to-severe allergic asthmatics. This observational study examines the “real world” effectiveness of omalizumab in this population.

**Methods:**

This is a one year open-label observational study that compared clinical outcomes including total oral corticosteroid use, exacerbation history, measures of quality of life and inflammation in patients with moderate-to-severe allergic asthma, who were prescribed omalizumab as part of their treatment with the year prior to therapy.

**Results:**

A total of 99 patients were enrolled at 25 sites in Canada. During the study period, the mean total annual OCS dose was reduced from 2301.5 mg (prednisone equivalents) in the year prior to omalizumab to 1130.0 mg (p<0.0001). There was a 71% reduction in asthma exacerbations and 56% of patients on omalizumab remained exacerbation free when compared to the year prior to study entry. Associated with this was reduced health care utilization. There were significant improvements in the Asthma Control Questionnaire (ACQ) and Asthma Quality of Life questionnaire (AQLQ) Patients with an elevated FeNO at baseline showed a better response to treatment. No new safety issues were identified during the study period.

**Conclusion:**

Our study demonstrates that in “real world” clinical practice, after initiating omalizumab, there is a reduction in total OCS use and exacerbation frequency in patients with moderate-to-severe allergic asthma. Patients on treatment reported improved asthma control and quality of life. FeNO may be a useful biomarker to identify patients who may benefit with omalizumab treatment.

## Introduction

Asthma is a chronic inflammatory airway disease characterized by variable airflow obstruction. Asthmatics are able to achieve control of their symptoms by limiting environmental exposures that may aggravate their asthma and by using medications such as inhaled corticosteroids (ICS) to reduce the inflammation associated with the disease.

However, there remains a population of asthmatics who, despite guideline-based treatment, continue to have poor control and experience acute exacerbations [1]. These patients often require oral corticosteroids (OCS) to control their disease. OCS are associated with a number of side effects including osteoporosis, cataracts and the potential for Cushing’s syndrome [2]. Since asthma is more severe in older adults [3], reducing the burden of OCS is of high importance.

Approximately 90% of asthma is allergic in nature [4]. Omalizumab is a biologic humanized IgE neutralizing antibody (anti-IgE) that binds to circulating IgE, prevents binding to the receptor thereby inhibiting the allergic-inflammatory cascade [5]. Omalizumab provides a non-steroidal treatment option for asthmatics with moderate to severe persistent allergic asthma, whose symptoms are inadequately controlled with high dose ICS [6]. Previously, omalizumab has been shown to reduce the number of asthma exacerbations, improve symptom severity, and improve the quality of life in such patients [7–9]. It is currently recommended in many asthma guidelines, including the Canadian guidelines [1], as part of a treatment algorithm [10].

The purpose of this study is to understand the ‘real world’ efficacy and safety of omalizumab in the treatment of patients with moderate-to-severe allergic asthma (the ASTERIX Observational study). The primary aim of this study was to evaluate the corticosteroid-sparing effect of omalizumab in these patients.

## Methods

### Study design

This was a pan Canadian, multicenter, 12-month, open-label single-arm study. At each institution, the research protocol was reviewed by the institutions ethics board (see below) and the study received institutional approval. This study is an observational study of patient related outcomes. The study medication is already approved for use in Canada, and the patients enrolled in the study were deemed to be appropriate for the use of omalizumab, as per the indications on the Canadian product monograph. Our study is assessing a real life response to the therapy. The study began in August 2012 (first patient, first visit) and completed in October 2014 (last patient, last visit). The study collected retrospective and prospective information from subjects who were to be prescribed omalizumab in routine clinical practice as per the Canadian Product Monograph [6]. Eligible study participants had inadequately-controlled asthma, defined as either: 1) having experienced two or more severe asthma exacerbations requiring treatment with OCS in the 12 months preceding study enrolment despite treatment with high dose ICS plus at least one other controller agent; or 2) requiring a maintenance dose of OCS for at least 3 months prior to study enrolment, independent of exacerbation history. During the study, subjects received treatment for their asthma and any acute asthma exacerbations as clinically indicated and as per usual medical practice.

Study eligibility criteria excluded subjects previously treated with omalizumab, subjects with a respiratory tract infection in the month prior to study start, current smokers or subjects with a smoking history of >10 pack-years, subjects with severe co-morbidities, or subjects who were pregnant or lactating.

Baseline assessments were completed prior to the first omalizumab injection and subjects were then assessed every three months during the study. At each assessment, pulmonary function was measured, medication use and exacerbation data were collected. The term “severe asthma exacerbation” was defined for this study as one that required treatment with systemic (oral or IV) corticosteroids and/or that resulted in a hospitalization or emergency department visit. Subjects also completed the Asthma Control Questionnaire (ACQ) and the Asthma Quality of Life Questionnaire (AQLQ). The ACQ is a seven-item questionnaire that is a validated assessment of asthma symptom control [11,12]. The AQLQ is a disease-specific, self-administered quality of life questionnaire [13] which can be used to evaluate the impact of asthma treatment over the prior 14 days.

Fractional exhaled nitric oxide (FeNO) was measured in a subset of enrolled subjects at 10 sites. Each of the 10 sites participating in this exploratory analysis had access to appropriate equipment for measuring FeNO, using standardized techniques and calibration procedures. Two FeNO tests were conducted on each subject at every study visit, and the reported value was the mean of 2 values that were within 10% of each other.

Retrospective data for a period of 12 months prior to the first omalizumab injection was collected from subject charts for comparison with data collected from the 12-month prospective study period. This data included: cumulative systemic corticosteroid dosage, frequency and severity of asthma exacerbations, symptom scores, quality-of-life data and health care utilization [14].

### Efficacy outcomes

The primary endpoint was the mean change in total annual OCS dose during the prospective treatment period as compared to the year prior to the start of omalizumab treatment. Secondary endpoints included the number (%) of subjects who reduced/stopped OCS use, the number (%) of subjects experiencing severe asthma exacerbations, the mean number of severe asthma exacerbations per subject, mean duration of severe asthma exacerbations, changes in ACQ, AQLQ and FeNO while receiving omalizumab as compared to baseline or the prior year. Health resource utilization data was also collected prospectively and compared to the prior year.

### Safety outcomes

Only serious adverse events were collected for study purposes.

### Statistical analysis/outcome measures

Statistical analyses were performed using the intent-to-treat (ITT) population. Descriptive statistics for continuous variables are presented with number of subjects, mean, standard deviation, median, minimum, and maximum. Comparisons between the prospective treatment period and retrospective data were performed using paired t-tests. Categorical variables were summarized with number and percentage of subjects. All hypotheses were based on two-sided tests using a 5% level of significance. The 95% confidence interval was calculated for the absolute difference between the treatment groups. Any other statistical tests were assessed at the 2-sided, 5% significance level.

The study protocol was approved by the institutional ethics board for each site, and all subjects (or parents/guardians when appropriate) provided written informed consent prior to any study-related evaluations. The following are the names of the ethics board/committee for each site involved in the study: Institutional Review Board Services (IRB Services), Aurora, Ontario; The Health Research Ethics Board (HREB)—Biomedical Panel, University of Alberta; Research Ethics Review Committee (RERC), Edmonton, Alberta; McGill University Health Center–Research Ethic Board (MUHC-REB), University of McGill; Comité d’Éthique à la Recherche–Centre Hospitalier de l’Université de Montréal (CER-CHUM), Centre Hospitalier de l’Université de Montréal; Comité d’Éthique à la Recherche–Institut Universitaire de Cardiologie et Pneumologie de Québec (CER IUCPQ), Institut Universitaire de Cardiologie et Pneumologie de Québec; University of British Columbia–Clinical Research Ethic Board (UBC CREB), University of British Columbia; University of British Columbia–Providence Health Care–Research Ethics Board (UBC-PHC REB, University of British Columbia.

## Results

A total of 99 subjects were enrolled at 25 sites in Canada. All subjects received at least one dose of omalizumab during the study period. Twenty-one subjects (21.2%) discontinued the study. The primary reasons for discontinuation were loss to follow-up (9 subjects), administrative reasons (5 subjects), adverse events (4 subjects) and subject no longer required study drug (3 subjects). Of the 4 subjects who discontinued due to adverse events, there were 3 serious and one non-serious event: 1 case of pregnancy, 1 case of anaphylactic reaction, 1 case of severe asthma and one subject who discontinued due to an unspecified non-serious adverse event.

Table 1 summarizes baseline characteristics. The subjects were predominantly female, white and with a mean age of 48. On average, they experienced 2.4 exacerbations in the year prior to starting treatment. Baseline medications are in keeping with current standards of asthma management.

**Table 1:** Demographics and baseline characteristics.

Baseline characteristics n = 99		
Age (years)	Mean (SD)	47.8 (13.8)
	Range	12–77
Gender, n (%)	Male	31 (31.3%)
	Female	68 (68.7%)
Race, n (%)	White	87 (87.9%)
	Black	5 (5.1%)
	Asian	5 (5.1%)
	Native American	2 (2.0%)
Weight (kg)	Mean (SD)	79.6 (19.7)
	Range	37–139
Duration of asthma (years)	Mean (SD)	20.9 (15.4)
Smoking history, n (%)	Never smoked	63 (63.6%)
	Ex-smoker	36 (36.4%)
FEV_1_ (L)	Mean (SD)	2.2 (0.8)
FEV1 (% predicted)	Mean (SD)	72.9 (20.1)
IgE levels (ng/mL)	Mean (SD)	668.1 (937.0)
Exacerbations in 12 months before baseline visit	Mean (SE)	2.4 (0.14)
	Mean (SE)	2.4 (0.14)
	Range	0.0–8.0
Asthma Medications taken at Visit 1*	Inhaled Corticosteroids	46 (46.5%)
	Long-Acting Bronchodilators	3 (3.0%)
	LABA/ICS (combination)	93 (93.9%)
	Leukotriene Receptor Antagonists	52 (52.5%)

LABA = Long Acting Bronchodilator; ICS = Inhaled Corticosteroid.

*A subject could have had more than one type of asthma medication.

All but one subject had documented prior systemic corticosteroid use. The majority used OCS periodically for the treatment of asthma exacerbations and 25% used a daily OCS maintenance dose for asthma management. When treated with omalizumab, the mean total annual OCS dose (expressed in prednisone equivalents) was reduced from 2301.5 mg (SE 374.3 mg) to 1130.0 mg (SE 307.5 mg) (p<0.0001) (Table 2). 70.8% of subjects either stopped or were able to reduce the dose of OCS by 40% or more.

**Table 2:** Mean change in total annual oral corticosteroid (OCS) dose [1] (ITT population).

	Retrospective Period	Prospective Period
N	96	96
Mean (SE)	2301.5 (374.3)	1130.0 (307.5)
Range	(0.0, 30780)	(0.0, 21840)
Retrospective—Prospective [2]		
• Difference (SE)	1171.5 (197.8)	
• 95% CI	[778.8, 1564.2]	
• p-value	<0.0001	

[1] Oral Corticosteroid values are converted to prednisone equivalents (expressed in mg).

[2] Paired t-test was used to assess period differences.

SE = standard error

Of the 96 subjects in the ITT population with available data, 54 patients (56.3%) remained exacerbation-free during the prospective period, as compared to only 4 patients (4.2%) in the retrospective period (Table 3). The overall exacerbation frequency was reduced by 70.8% during the study period, with a mean of 0.8 severe exacerbations/patient compared to 2.4/patient in the year prior (p<0.0001). Additionally, the mean duration of severe asthma exacerbations was decreased from 39.1 to 10.9 days, (p<0.0001).

**Table 3:** Number (%) of subjects experiencing severe asthma exacerbations [1] and change from baseline health resource utilization (ITT population).

		Retrospective period	Prospective period
Severe asthma Exacerbation	N	96	96
	No	4 (4.2%)	54 (56.3%)
	Yes	92 (95.8%)	42 (43.8%)
Number of emergency department (ED) visits	N	95	96
	Mean (SE)	1.2 (0.1)	0.2 (0.1)
	Median	1.0	0.0
	Range	(0.0, 7.0)	(0.0, 5.0)
	Retrospective—Prospective [2]		
	• Difference (SE)	1.1 (0.2)	
	• 95% CI	[-0.8,1.4]	
	• p-value	<0.0001	
Number of hospitalizations	N	96	96
	Mean (SE)	0.4 (0.1)	0.1 (0.1)
	Median	0.0	0.0
	Range	(0.0, 5.0)	(0.0, 4.0)
	Retrospective—Prospective [2]		
	• Difference (SE)	0.3 (0.1)	
	• 95% CI	[-0.1, 0.5]	
	• p-value	0.0081	
Number of unscheduled health professional visits	N	87	95
	Mean (SE)	3.1 (0.4)	0.8 (0.2)
	Median	3.0	0.0
	Range	(0.0, 20.0)	(0.0, 8.0)
	Retrospective—Prospective [2]		
	• Difference (SE)	2.4 (0.4)	
	• 95% CI	[1.7, 3.1]	
	• p-value	<0.0001	
Number of days in hospital (ED+ICU+Other)	N	96	96
	Mean (SE)	1.8 (0.3)	0.6 (0.3)
	Median	0.0	0.0
	Range	(0.0, 18.0)	(0.0, 22.0)
	Retrospective—Prospective [2]		
	• Difference (SE)	1.3 (0.4)	
	• 95% CI	[0.5, 2.0]	
	• p-value	0.0010	

[1] A severe asthma exacerbation had to fulfil at least one of the following criteria: 1) requiring treatment with systemic (oral or IV) corticosteroids OR 2) resulting in hospitalization or requiring an emergency department visit. Courses of corticosteroids separated by 1 week or more were treated as separate severe exacerbation events

[2] Paired t-test was used to assess period differences.

Omalizumab treatment resulted in improved asthma control, as shown through significant improvements in the mean ACQ total score, from 2.7 at baseline to 1.9 at month 4 (p<0.0001). This improvement was maintained until the end of the study (Table 4). In parallel, the AQLQ score improved from an average of 3.9 at baseline to 4.8 at month 4 (p<0.0001) (Table 5) and remained stable for the remainder of the study. The domain scores all improved by a similar magnitude.

**Table 4:** Change from baseline in Asthma Control Questionnaire (ACQ) total score [1] during the prospective period (ITT population).

	Baseline	Month 4	Month 8	Month 12
N	96	92	76	86
Mean (SE)	2.7 (0.1)	1.9 (0.1)	1.8 (0.1)	1.9 (0.1)
Median	2.6	1.8	1.7	1.8
Range	(0.0, 5.6)	(0.0, 4.6)	(0.0, 4.0)	(0.0, 4.7)
Baseline—Treatment				
• Difference (SE)		0.7 (0.1)	1.0 (0.1)	0.8 (0.1)
• 95%CI		[0.5, 1.0]	[0.8, 1.2]	[0.5, 1.1]
• p-value		<0.0001	<0.0001	<0.0001

[1] Each question is equally weighted and the ACQ score is the mean of the 7 questions with an average value between 0 (totally controlled) and 6 (severely uncontrolled). Paired t-test was used to assess differences between baseline and treatment values.

**Table 5:** Change from baseline in Asthma Quality of Life Questionnaire (AQLQ) total scores during treatment with omalizumab (ITT population).

	Baseline	Month 4	Month 8	Month 12
**Average Overall Score**				
N	96	92	76	86
Mean (SE)	3.9 (0.1)	4.8 (0.1)	5.0 (0.1)	4.8 (0.2)
Median	3.9	4.9	5.1	4.9
Range	(1.4, 6.7)	(1.8, 6.8)	(1.8, 6.9)	(1.6, 6.9)
Baseline—treatment [1]				
• Difference (SE)		-0.9 (0.1)	-1.2 (0.1)	-1.0 (0.2)
• 95% CI		[-1.2, -0.6]	[-1.5, -0.9]	[-1.3, -0.7]
• p-value		<0.0001	<0.0001	<0.0001

[1] Paired t-test was used to assess differences between baseline and treatment values.

FeNO was measured on 48 subjects during the study period. Table 6 summarizes the change in FeNO measurements over the course of the study period. Mean FeNO at baseline was 47.3 ppb (SE 5.7) and was significantly reduced by 12 months to 37.1 ppb (SE 4.6, p = 0.0258). Subjects were further divided into high (≥19.5 ppb) and low (<19.5) baseline FeNO groups for analysis purposes (Table 7) [15]. There was a higher percent reduction in total annual OCS dose and annual number of severe asthma exacerbations in the high FeNO subgroup than in the Low FeNO group. In the high FeNO group, the annual OCS dose decreased by 75% and exacerbations were reduced by 82%.

**Table 6:** Change from baseline in Forced Exhaled Nitric Oxide (FeNO) mean values [1] during the prospective period (ITT / FeNO population).

	Baseline	Month 4	Month 8	Month 12
N	48	46	47	49
Mean (SE)	47.3 (5.7)	39.1 (5.0)	38.0 (6.0)	37.1 (4.6)
Median	39.3	26.5	23.5	22.0
Range	(6.5, 173.5)	(5.5, 161.5)	(5.0, 238.5)	(5.0, 140.0)
Baseline—treatment [2][3]				
• Difference(SE)		7.6 (4.0)	9.0 (4.8)	9.9 (4.3)
• 95% CI		[-0.5, 15.7]	[-0.6, 18.6]	[1.2, 18.5]
• p-value		0.0660	0.0642	0.0258

[1] The FeNO values (in ppb) used for the summary were the mean of 2 values obtained at each visit.

[2] Paired t-test was used to assess differences between baseline and treatment values.

[3] This analysis was conducted on a subset of subjects at 10 pre-defined sites.

Note: Last observation forward methods (LOCF) were used for missing data.

**Table 7:** Percent change in total annual OCS dose and number of severe asthma exacerbations in Low/High FeNO baseline subgroups from baseline to end of prospective period (ITT population).

		% change in total annual OCS dose [1]	% change in total annual number of severe asthma exacerbations [2]
High FeNO [3]	N	35	35
	Mean (SE)	-75.21 (5.5)	-81.8 (4.9)
	Median	-100.0	-100.0
	Range	(-100.0, 0.3)	(-100.0, 0.0)
Low FeNO [3]	N	13	13
	Mean (SE)	-27.6 (25.0)	-47.4 (18.4)
	Median	-52.6	-66.7
	Range	(-100.0, 182.2)	(-100.0, 100.0)

[1] Oral corticosteroid values were converted to prednisone equivalents (mg).

[2] Percent change was defined as 100*(Prospective—Retrospective)/Retrospective.

[3] Low FeNO = FeNO measurement <19.5 ppb at baseline; High FeNO = FeNO measurement ≥19.5 ppb.

Note: Two subjects had no severe asthma exacerbation during the Retrospective period but had one during the Prospective period. Therefore, % change could not be computed for these subjects.

Table 3 summarizes the change in healthcare resource utilization during the study period. The mean number of Emergency Department visits (0.2 ± 0.1 vs 1.2 ± 0.1, p<0.0001), hospitalizations (0.1 ± 0.1 vs 0.4 ± 0.1, p = 0.0081) and unscheduled healthcare professional visits (0.8 ± 0.2 vs 3.1 ± 0.4, p<0.0001) were all significantly reduced during the treatment follow up period. In addition, hospitalized subjects had a significantly reduced length of stay (0.6 ± 0.3 vs 1.8 ± 0.3 days; p<0.0010).

### Safety

In the study database, nine subjects experienced at least one SAE (Serious Adverse Event) but only one subject experienced an SAE considered by the investigator to be related to study drug. That subject had three events—dizziness of moderate intensity on the first day of treatment and 2 anaphylactic reactions of severe intensity. Omalizumab was permanently discontinued for this subject. The subject was reported to have completely recovered from these events. Table 8 summarizes all SAEs reported during the study. No safety concerns were raised from these SAEs. One subject became pregnant and was discontinued from the study.

**Table 8:** All Serious adverse events.

System Organ Class	Omalizumab (N = 99)
**Preferred Term**	n (%)
**Infections and Infestations**	
Abscess	1 (1%)
Device related infection	1 (1%)
Post procedural infection	1 (1%)
Staphylococcal infection	1 (1%)
**Injury, poisoning and procedural complications**	
Anaemia postoperative	1 (1%)
Procedural haemorrhage	1 (1%)
Wrist fracture	1 (1%)
**Cardiac disorders**	
Cardiac arrest	1 (1%)
**Gastrointestinal disorders**	
Large intestinal obstruction	1 (1%)
**Immune system disorders**	
Anaphylactic reaction	1 (1%)
**Nervous system disorders**	
Dizziness	1 (1%)
**Pregnancy, puerperium and perinatal conditions**	
Pregnancy	1 (1%)
**Renal and urinary disorders**	
Renal impairment	1 (1%)
**Respiratory, thoracic and mediastinal disorders**	
Asthma	1 (1%)

## Discussion

Among the asthmatic population, the prevalence of severe uncontrolled asthma is approximately 10% [16,17]. These patients have a reduced quality of life and poor lung function. For many of them, systemic corticosteroids are routinely used to manage the disease, and as a result, they are prone to a variety of short and long-term adverse effects [2], which may further add to the clinical burden of this disease. In randomized clinical trials omalizumab, through its non-steroidal mechanism of action of binding free IgE molecules and preventing activation of the inflammatory cascade, has been shown to reduce asthma exacerbations and thereby reduce the need for oral corticosteroids [8,9]. However, until now the Canadian real-life effect has not been assessed. The present study was designed to evaluate the “real life” clinical benefit of omalizumab as add-on treatment for patients with poorly controlled moderate-to-severe allergic asthma, as per Canadian guidelines [1].

Our results demonstrate that patients treated with omalizumab reduced their total oral corticosteroid dosing during the treatment period, when compared with the year prior. Furthermore, 50% of the patients were able to discontinue use of daily OCS during the study. There are significant benefits to this, as long-term OCS use is associated with many side effects negatively affecting a patient’s overall health. These include increased risk of infections, hypertension, cataracts, impact on bone mineral density and the potential to lead to diabetes or affect diabetic control [2].

Prevention of asthma exacerbations is an important outcome of asthma treatment [1, 10]. Our study demonstrated a significant reduction in the frequency, severity and duration of asthma exacerbations. Although mortality due to asthma has decreased significantly, the morbidity and costs associated with asthma exacerbations remain elevated [16,18,19]. In our study, we saw a 71% reduction in total asthma exacerbations along with reduced health care utilization. In several studies, the improved outcomes we observed has resulted in a cost benefit favoring use of omalizumab, especially in those with severe exacerbations requiring hospitalizations [20,21].

FeNO is considered to be a non-invasive marker for airway inflammation [22] and has been utilized as a surrogate marker for the presence of eosinophilic airway dysfunction. In this study, we observed a significant reduction in FeNO levels over the 1-year study period. The FeNO decrease occurred despite reduced OCS exposure, supporting the anti-inflammatory effect of omalizumab. Furthermore, the clinical benefits of omalizumab were more pronounced in patients who had high FeNO levels at baseline. There was a 75% reduction in annual OCS use and an 82% reduction in exacerbation rates in patients with a FeNO ≥19.5 ppb. This supports the findings of Hanania et al.[15] who demonstrated a similar finding in the EXTRA study. FeNO, if used as part of the clinical assessment, may further assist in identifying an omalizumab responder, which is of benefit for patient and physician.

According to the Canadian asthma management continuum [1], improved quality of life (QoL) and symptom control are important goals of treatment. The subjects enrolled in this study had poor QoL and suboptimal control of their asthma at baseline, as measured by the ACQ and AQLQ. The AQLQ is a disease-specific questionnaire which measures the impact of asthma on symptoms, activity limitation, emotional function and environmental stimuli [13]. In this study, omalizumab treatment led to a significant improvement in AQLQ scores. The improvements were seen early, by month 4, and were maintained throughout the assessment period. In parallel, the ACQ demonstrated a similar early significant improvement in asthma symptoms and these were also maintained over the course of the study. It is important to highlight that all the improved outcomes observed during the study occurred in the setting of reduced OCS exposure, which has its own clinical merit and further supports the efficacy of omalizumab.

Although only 5–10% of asthmatics have severe disease, they account for more than 50% of asthma-related total health costs [18,23]. Our results show that adding omalizumab to the therapeutic regimen of these severe asthmatics significantly reduced overall health care utilization. Reductions in healthcare utilization with omalizumab have been previously reported [7,9,24–26], and it is encouraging, that in “real life” application, this continues to be seen.

ASTERIX is an observational, open-label study and the results are consistent with published randomized clinical trials and other “real life effectiveness” studies [7–9,27], however this is the first report in a Canadian population and the first to report on the use of FeNO in “real life” Canadian clinical practice. Recently both a systematic review and meta-analysis assessing “real life effectiveness” studies of omalizumab have been published [28, 29]. The outcomes assessed and reported in those studies were similar to ours, and show that in different populations, there is a “real life effectiveness” of omalizumab in improving the outcomes for patients with severe asthma.

The results of our study in a Canadian population adds to the current literature in several capacities. First, there are inherent differences in the Canadian population than the ones studied in the systematic review and meta-analysis, as those studies were primarily European. Second, because health care systems vary between countries, the medical treatment a patient receives prior to the initiation of omalizumab may vary, and thereby affect the outcomes of previous reports. Third, demonstrating efficacy in the Canadian severe asthma population will be helpful to payers of the health care system in Canada, both private and public, who may request evidence of efficacy in order to fund this medication. Lastly, unique to our study was the measurement of FeNO in a sub-set of the study population. The impressive response to omalizumab in patient’s with high FeNO (>19.5 ppb) has not been reported in other “real life” studies. Based on both the EXTRA and Asterix study, clinicians may want to consider measuring FeNO to provide more confidence in the decision to add on omalizumab to current therapy in the uncontrolled severe asthma patient.

There are several limitations to this study. First, it is an open-label study and there is no control group for comparison. Instead, treatment effectiveness was assessed in comparison to the previous year or to patient status at baseline (AQLQ, ACQ). Second, it could be considered a weakness that some of the study data (exacerbations, OCS use and acute care visits) was collected retrospectively. However, subjects with this severity of asthma tend to be followed closely by their physicians so that there is a good record of treatment in the patient chart. If anything, some retrospective events might not have been reported and the results may therefore underestimate the treatment effect.

## Conclusion

In a “real world” observational study, patients with moderate-to-severe allergic asthma who are poorly controlled despite optimized therapy with high dose ICS and another controller medication, benefit from treatment with omalizumab by reducing oral steroid use, experiencing reduced exacerbation frequency and showing improved quality of life and asthma control.
